# Assessing the evolution of infectious disease preparedness among a province with poor economy in China in the wake of COVID-19

**DOI:** 10.3389/fpubh.2025.1472331

**Published:** 2025-05-26

**Authors:** Huaying Liu, Qun Liang, Chen Lai, Huiping Liang

**Affiliations:** ^1^Department of Medicine, Guangxi Health Science College, Nanning, China; ^2^Guangxi University of Chinese Medicine, Nanning, China

**Keywords:** physicians, grassroots hospital, infectious diseases, COVID-19, training needs

## Abstract

**Introduction:**

To identify the factors influencing changes in knowledge and experience of infectious disease prevention and control among physicians in Guangxis grassroots hospitals before and after the COVID-19 pandemic, thereby offering insights to enhance emergency response capabilities, disease prevention and control proficiency, and the overall effectiveness of grassroots medical institutions during epidemics.

**Methods:**

Utilizing random sampling, we conducted questionnaire surveys among primary care physicians from Guangxis primary medical institutions in June 2019 and October 2022, and analyzed the data with SPSS software.

**Results:**

Post-COVID-19, there was a significant increase in the scores for theoretical knowledge (77.10 ± 14.83 vs. 63.10 ± 15.11, *p* < 0.001) and understanding of infectious disease regulations (54.78 ± 15.94 vs. 50.85 ± 12.52, *p* = 0.001) among grassroots hospital physicians compared to pre-COVID-19 levels, albeit with room for further improvement. The participation rate in emergency treatment of infectious diseases rose to 63.51% after COVID-19, from 58.33% of physicians lacking infectious disease training before the pandemic, which decreased to 35.60% post-pandemic. Currently, “community family medical observation” is the most sought-after training content among Guangxis grassroots physicians (31.25%).

**Discussion:**

The capacity for infectious disease prevention and control among Guangxis grassroots hospital physicians has significantly improved following COVID-19. The high engagement in related training and emergency response efforts reflects a strong sense of professional identity and commitment. Ongoing, needs-based infectious disease training is recommended to ensure that grassroots medical staff can optimally contribute to the management of infectious disease emergencies.

## 1 Introduction

Since the end of 2019, COVID-19 has continued to pose a significant global health challenge (1). The capacity for infectious disease prevention and control among frontline medical staff is pivotal for effective disease management and patient care. Grassroots hospital physicians, as the first line of defense, are entrusted with the critical mission of disease prevention and public health protection at the community level. As outlined in the National Basic Public Health Service Specification (Third Edition) of China, these institutions are mandated to support contact tracing, epidemiological investigations, sanitation of contaminated areas, and vector control activities (2). Their foundational role in the COVID-19 response has become increasingly evident, with growing responsibilities and the gradual impact of strengthened infrastructure.

Guangxi, a province in China with relatively lagging economic and healthcare development, faces distinct challenges within its grassroots medical facilities. These include personnel shortages, lower educational attainment, professional credentialing issues, inadequate infrastructure, and deficiencies in emergency preparedness and staff training mechanisms (3). According to the economic report of China Government Network in 2023, Guangxi’s total gross domestic product (GDP) in 2023 ranks 19th in the country (19/31), with the second lowest growth rate (30/31). In 2023, Guangxi’s GDP reached just 2.7 trillion yuan, while its neighboring province, Guangdong to the east, ranked first with a GDP of 13.56 trillion yuan (4).

As a province with poor economy, Guangxi does not have the strong guarantee of primary healthcare like other economically developed provinces in China. In the face of the outbreak of acute infectious diseases, Guangxi, which is relatively backward in all aspects, has established corresponding guarantee mechanisms and sufficient capacity for epidemic prevention and control after COVID-19. We hypothesize that these robust guarantee mechanisms can enhance the infectious disease prevention and control capabilities of grassroots physicians in Guangxi, while also reinforcing their mastery of relevant knowledge at the primary care level. Examining the evolution of infectious disease management skills among grassroots physicians in Guangxi—an economically underdeveloped region—can provide valuable insights into broader trends and the current landscape of public health in China.

Our team has been responsible for the ongoing training and evaluation of grassroots physicians in Guangxi, with infectious disease prevention and control consistently included as a core component of our training programs. The research team is fully dedicated to studying infectious disease management at the community level. Even before the COVID-19 outbreak in late 2019, this work—including our survey research—had been carried out continuously and persistently, both before and during the pandemic. Now, we aim to assess the effectiveness of policy implementation before and after COVID-19. Our prior research indicated that before the COVID-19 pandemic, primary healthcare institutions in Guangxi grappled with a range of issues, including a focus on single diseases, a dearth of skilled teams, and an imbalance in multidisciplinary development. The proficiency of medical staff in peripheral disciplines, notably infectious disease prevention and control, as well as their capacity to manage public health emergencies, were areas identified for improvement (5, 6). In the wake of COVID-19, assessing the current state of knowledge and competencies in infectious disease management among grassroots physicians is imperative. This includes evaluating improvements, training modalities, and their adequacy in addressing local infectious disease control demands.

The present study aims to delve into the competencies, experiences, training status, and needs regarding infectious disease prevention and control among Guangxi’s grassroots hospital physicians before and after the COVID-19 pandemic. We want to analyze the current situation of epidemic prevention and control capabilities in underdeveloped regions of China after COVID-19, and to provide reference opinions for other underdeveloped regions. The findings are intended to inform strategies for enhancing knowledge, improving response levels to infectious disease emergencies, refining preventive measures, and crafting efficacious epidemic response protocols.

## 2 Data and methods

### 2.1 Study design

We employed a stratified random sampling method. First, township health centers in Guangxi were categorized by county. Then, proportional random sampling was conducted within each stratum to ensure regional representativeness. According to the sampling results, a longitudinal questionnaire survey was conducted among a cohort of primary care physicians from 50 grassroots hospitals in Guangxi, sampled randomly in June 2019 and re-surveyed in October 2022. To avoid bias, both surveys were conducted by the same group of people, and when the research subjects were surveyed twice, their educational background and professional title remained consistent. Data from research subjects who only participated in one survey need to be excluded. Participants met the following criteria: they were licensed clinicians engaged in frontline medical work at primary healthcare institutions in Guangxi; possessed at least 1 year of professional experience; voluntarily consented to participate in the study; completed both survey instances. Our institution serves as a training base for grassroots physicians in Guangxi. To better understand trainees’ educational needs, we conduct questionnaire surveys. In order to better understand the educational needs of the students, we conducted a questionnaire survey. This research was approved by the Ethics Committee of Guangxi Health Science College. All participating students were fully informed and provided written consent before taking part (Approval No. Z-A20221009).

### 2.2 Research methods

#### 2.2.1 Survey instrument

The survey questionnaire was divided into three sections (as shown in Appendix):

Demographic Information: This included gender, age, workplace, specialty, duration of practice, educational attainment, professional qualifications, employment status, and whether their current or previous role involved infectious disease management.
Knowledge Assessment: A section dedicated to evaluating the physicians’ mastery of infectious disease prevention and control knowledge, which was divided into two components: ① Regulatory Knowledge: Including the classification, reporting, and public health response to infectious diseases. ② Prevention and Control Competence: Assessing both foundational theoretical knowledge and practical operational skills in infectious disease management. Scores were calculated as the ratio of correct answers to the total possible points, with a percentage scale where <60% indicated low proficiency, 60–79% indicated medium proficiency, and ≥80% indicated high proficiency. To minimize bias, we implemented the following measures: designed equivalent question sets that maintained consistent testing objectives while varying question phrasing; ensured comparable difficulty levels across all questions; maintained uniform question structure throughout; selected questions from the standardized Chinese Physician Licensing Exam question bank to enhance objectivity and randomization; incorporated anchor questions to calibrate difficulty; engaged four expert professors to review and pretest the questions, ensuring both content validity and appropriate formatting; the survey questionnaire demonstrated satisfactory internal consistency across all subscales (Cronbach’s *α* > 0.7).
Professional Experience and Training Needs: Encompassing current roles in infectious disease prevention and control, experience with emergency response, training history, willingness to train, and expressed training needs.

#### 2.2.2 Data collection protocol

The surveys were administered by a consistent team of investigators, following a standardized protocol to ensure uniformity. Physicians completed paper questionnaires anonymously, which were collected on-site. Data entry was performed double-blind by two independent operators, with an additional overseer ensuring data integrity and accuracy.

#### 2.2.3 Statistical analysis

Data analysis was conducted using SPSS version 22.0. Continuous variables between groups were compared using analysis of variance (ANOVA), binary variables were assessed with the Chi-square test, and ordered categorical variables were evaluated with the Kruskal-Wallis test. Multivariate logistic regression was employed for correlated analyses. Statistical significance was set at *p* < 0.05.

## 3 Results

### 3.1 Baseline characteristics and partial answer situation of all the physicians

The survey was conducted across a random sample of fifty grassroots hospitals in Guangxi. Initially, six hundred participants were targeted, from which 599 questionnaires were collected, yielding 597 valid responses, reflecting an effective response rate of 99.67%. Post-COVID-19, a follow-up survey of the same cohort was carried out, resulting in 590 valid responses out of 597, with an effective rate of 98.83%. Demographic data of the respondents are as follows: 270 males (48.21%) with an average age of 32.7 years, and 320 females (54.24%) with an average age of 28.0 years. Educational attainment included 361 individuals (61.19%) with a college degree or higher, and 229 (38.81%) with a technical secondary school degree or lower. Professional titles were distributed between 280 respondents (47.46%) holding intermediate or higher titles, and 310 (52.54%) with primary titles. Notably, none of the respondents specialized in infectious diseases (as shown in Table 1).

**Table 1:** Baseline characteristics and partial answer situation of all the physicians.

Characteristics	All (590)
Age, year	27 (35)
Men, *n* (%)	270 (48.21%)
Educational background, *n* (%)	
College degree or higher	361 (61.19%)
Technical secondary school degree or lower	229 (38.81%)
The title of a technical or professional post, *n* (%)	
Intermediate or higher titles	280 (47.46%)
Primary title	310 (52.54%)
Pre-COVID-19, *n* (%)	
Had not received any training related to infectious diseases	344 (58.31%)
Difficulties encountered in the prevention and control of infectious diseases, *n* (%)	
Inspection methods for infectious diseases	396 (67.12%)
Differential diagnosis	350 (59.32%)
Therapeutic medication	337 (57.12%)
Post-COVID-19, *n* (%)	
Had not received any training related to infectious diseases	210 (35.60%)
Participated in the prevention and control of infectious diseases	427 (72.37%)
Grassroots physicians need to provide health education to the general public	490 (94.78%)
Difficulties encountered in the prevention and control of infectious diseases, *n* (%)	
Differential diagnosis	404 (68.47%)
Therapeutic medication	338 (57.29%)
Inspection methods for infectious diseases	331 (56.1%)
Pre diagnosis and triage of infectious diseases	311 (52.71%)
Isolation ward’s ability to prevent and control infectious diseases	207 (35.08%)
Training needs, *n* (%)	
Community family medical observation	210 (35.60%)
Science popularization preaching	179 (30.34%)
Concentrated medical observation in quarantine are	145 (24.58%)

### 3.2 Comparative analysis of infectious disease prevention and control abilities

The comparative analysis of the abilities of Guangxi’s grassroots physicians in infectious disease prevention and control, pre-and post-COVID-19, is detailed in Table 2. Post-COVID-19, the scores reflecting the doctors’ understanding of infectious disease laws and regulations were significantly elevated compared to pre-COVID-19 scores (54.78 ± 15.94 vs. 50.85 ± 12.52, *p* = 0.001). Similarly, there was a marked improvement in the theoretical knowledge scores related to infectious disease prevention and control (77.10 ± 14.83 vs. 63.10 ± 15.11, *p* < 0.001).

**Table 2:** Analysis of the ability of Guangxi grass-roots physicians to prevent and control infectious diseases before and after COVID-19.

Item	Pre-COVID-19	Post-COVID-19	*p*
Laws and regulations of infectious disease	50.85 ± 12.52	54.11 ± 15.94	0.001
Theoretical knowledge of infectious disease	63.10 ± 15.11	77.10 ± 14.83	0.001

### 3.3 Univariate analysis of influencing factors

Univariate analysis of grassroots physicians’ mastery of theoretical knowledge related to infectious diseases after COVID-19 is presented in Table 3. The analysis revealed that professional title (value = 4.172, *p* = 0.041) and experience in infectious disease prevention and control (value = 3.968, *p* = 0.048) significantly influence the theoretical knowledge scores of grassroots physicians. However, as detailed in Table 4, factors such as age, gender, education level, workplace, and training experience did not significantly affect the ability to understand infectious disease laws and regulations.

**Table 3:** Univariate analysis of grassroots physicians’ mastery of theoretical knowledge related to infectious diseases after COVID-19.

Item	Value	*p*
Gender	0.948	0.342
Age	0.283	0.595
Title	4.172	0.041
Licensed place	3.873	0.568
Education	0.177	0.697
Experience in infectious disease prevention and control	3.968	0.048

**Table 4:** Univariate analysis of the ability of grassroots physicians to master infectious disease laws and regulations after COVID-19.

Item	Value	*p*
Gender	0.461	0.516
Age	1.930	0.165
Title	1.328	0.266
Licensed place	11.634	0.678
Education	0.088	0.776
Experience in infectious disease prevention and control	0.044	0.839

### 3.4 Multivariate analysis of theoretical knowledge mastery

A multivariate linear regression analysis was conducted with the total score of grassroots physicians’ theoretical knowledge of infectious disease prevention and control as the dependent variable and the significant factors (*p* < 0.05) from the single factor analysis as independent variables. The results, displayed in Table 5, indicate a positive correlation between the professional title of primary care doctors and their theoretical knowledge scores (OR = 1.544, *p* = 0.037, 95% CI: 1.027–2.320).

**Table 5:** Multivariate analysis of the ability of grassroots physicians to master the theoretical knowledge of infectious disease prevention and control.

Item	B	OR	95%CI		*p*
Title	0.434	1.544	1.027	2.320	0.037
Contact history	−0.448	0.639	0.398	1.026	0.064

### 3.5 Participation in infectious disease prevention and control activities

Prior to COVID-19, 58.31% (344 individuals) of Guangxi’s grassroots physicians reported not having received training related to infectious diseases. This figure decreased to 35.60% (210 individuals) post-COVID-19 outbreak. Of the 590 surveyed doctors, 72.37% (427 individuals) participated in infectious disease prevention and control activities following the outbreak. Pre-COVID-19, the most significant challenges and areas for improvement identified by grassroots physicians were diagnostic methods (67.12%), differential diagnosis (59.32%), and knowledge of therapeutic drugs (57.12%). Post-COVID-19, the primary difficulty in daily work was managing infectious disease prevention and control within isolation wards, cited by 35.08% of doctors. The top training needs identified were community family medical observation (35.60%), public health education (30.34%), and protocols for centralized medical observation in isolation wards, including safety protection and waste management (24.58%). After the COVID-19 outbreak, 94.78% (490 individuals) of grassroots physicians felt a need for public health science popularization, while only 1.35% (7 individuals) saw no need for such initiatives (as shown in Table 1).

## 4 Discussion

### 4.1 Interpretation of findings

Utilizing questionnaire analysis, this study examines the shifts in the infectious disease prevention and control capabilities of grassroots physicians in Guangxi before and after the COVID-19 pandemic, as well as their current training requirements. The results indicate a significant enhancement in the theoretical knowledge scores and understanding of infectious disease laws and regulations among grassroots physicians post-COVID-19. However, the overall proficiency remains modest, suggesting a need for continued educational initiatives. Notably, “Community family medical observation” emerged as the most sought-after training module, highlighting a specific area for improvement (31.25%) (7, 8).

The analysis suggests that the COVID-19 pandemic has catalyzed an improvement in the infectious disease prevention and control capabilities of grassroots physicians in Guangxi. This upswing reflects the impact of targeted measures, indicating that grassroots physicians are now better equipped to manage similar public health challenges in the future (9). Post-COVID-19, there has been a marked increase in the mastery of infectious disease laws, regulations, and prevention and control abilities among Guangxi’s grassroots physicians. The statistical analysis revealed a significant disparity in the pre-COVID-19 baseline knowledge compared to the post-COVID-19 period. The primary care setting prior to COVID-19 was predominantly focused on chronic disease management and maternal and child health, with less emphasis on infectious diseases. The lack of specialized fever clinics and infectious disease departments may have contributed to a lower recognition rate of infectious diseases by doctors (10, 11).

Furthermore, the proportion of grassroots physicians in Guangxi who had not received infectious disease training dropped from 58.33% before the pandemic to 35.60% after the outbreak. This decrease is likely attributed to the proactive organization of epidemic prevention and control efforts and the implementation of various infectious disease training programs at different administrative levels. Survey data corroborate this trend, showing an increase in the participation of grassroots physicians in infectious disease-related training from 41.67% pre-COVID-19 to 64.40% post-COVID-19, with 72.37% engaging in infectious disease prevention and control activities. This increase in training participation and the organized efforts to enhance the skills of grassroots medical staff have yielded positive outcomes, significantly bolstering their infectious disease prevention and control competencies. These developments are closely tied to the series of regulations and policies implemented by the Chinese government following COVID-19. During the initial phase of the pandemic in early 2020, the Chinese government promptly issued policy directives mandating enhanced training for all primary healthcare workers and accelerated improvements in grassroots prevention capabilities. The Guangxi regional government responded swiftly, implementing various policy requirements within 1 month of receiving the central government’s notice. In 2024, Guangxi introduced additional measures to strengthen the allocation of public health personnel at the grassroots level, significantly improving primary care physicians’ capabilities in public health emergency response, infectious disease treatment, intervention, and prevention. China’s multi-level government system demonstrates remarkable policy implementation efficiency and institutional specificity in this regard. These coordinated efforts have created substantial training opportunities for grassroots physicians and dramatically enhanced their infectious disease management capabilities. This phenomenon extends beyond China. The COVID-19 pandemic has triggered global transformations in epidemic prevention strategies. The World Health Organization (WHO) now promotes community-based prevention approaches, offering multilingual distance learning courses and standardized training modules for primary healthcare workers (12). In the United States, the Federally Qualified Health Centers (FQHCs) Enhancement Program has increased federal funding to bolster infectious disease response capabilities in community clinics serving low-income populations. The program facilitates knowledge transfer through remote video training and case discussions connecting primary care providers with university and specialty hospital experts. The pandemic has underscored the critical role of primary healthcare institutions in global epidemic response (13). Countries worldwide are now implementing diverse measures to strengthen primary care practitioners’ capabilities, better preparing healthcare systems for future public health emergencies.

Our study identified a significant correlation between the professional title level of grassroots physicians and their mastery of infectious disease knowledge. Single factor analysis highlighted both professional title and prior experience with infectious diseases as influential factors in theoretical knowledge scores. Multiple linear regression analysis further substantiated a positive association between the professional title of grassroots physicians and their theoretical knowledge scores (OR = 1.544, *p* = 0.037, 95% CI: 1.027–2.320). These findings echo previous research by Kong et al. (14) and Liu et al. (15), which suggests that longer practice durations and higher professional titles are indicative of greater exposure to and understanding of infectious disease knowledge, thereby enhancing emergency response capabilities. A cross-sectional study on primary healthcare institutions in Bangladesh evaluated the knowledge and practices of healthcare personnel in areas such as personal hygiene, medical device handling, waste management, and triage. The results showed that the professional titles and educational levels of medical and health personnel were significantly correlated with their knowledge in infection prevention and control (16).

However, the present survey revealed that the professional title level and educational background of grassroots physicians in Guangxi are generally low, with a significant proportion (38.81%) holding education levels at the technical secondary school level or below, and only 47.46% holding intermediate or higher professional titles. The absence of infectious disease specialists among the surveyed doctors (0 out of 590) underscores a critical gap in the capacity of grassroots medical institutions to engage in infectious disease prevention and control. For China, grassroots medical institutions in economically underdeveloped areas severely lack physician resources (14). A study in China shows that from 2016 to 2020, the high concentration areas of health personnel, health technicians, practicing (assistant) physicians, and registered nurses in township health centers in China were mostly in the central and eastern regions. The reasons may as follows: firstly, the eastern region of China has superior conditions in various aspects, such as sufficient funds, technology and other resources, which provide important guarantees for the career development of health talents, while the western region has relatively backward economic development and weaker attraction to health talents. Secondly, personnel have the characteristic of mobility, and the personnel allocation in various regions is influenced by the level of health resource allocation in surrounding developed provinces. More talents tend to go to areas with better conditions. From this, it can be seen that western regions of China, such as Guangxi, still need to continuously make up for their own shortcomings, improve their attractiveness to high-level talents, and thus enhance their epidemic prevention and control capabilities (17). As previously noted, Guangxi’s 2023 GDP reached only 2.7 trillion yuan (ranking 19th nationally) in the post-COVID era (4). This economic constraint has created significant challenges in funding grassroots medical teams, particularly for building infectious disease prevention and control capabilities at primary healthcare institutions. Despite these financial limitations, the government has prioritized strengthening grassroots disease prevention capacity through targeted funding and policy reforms in the pandemic’s aftermath. Notably, in 2024 Guangxi implemented reforms to the professional certification system for primary care physicians, including streamlined senior title qualifications. Physicians meeting specific requirements can now obtain senior professional titles after ten years of service without undergoing traditional evaluation processes. These policy adjustments serve multiple purposes: retaining skilled physicians in community healthcare settings, enhancing professional competencies, and ultimately improving grassroots medical institutions’ capacity for comprehensive disease prevention and control.

The COVID-19 pandemic persists globally, with the virus exhibiting increased transmissibility due to mutations (18). The evolution of prevention measures has steered epidemic control into a “new stage,” shifting the focus from “infection prevention” to “health protection and severe disease prevention.” It is imperative to excel in rural COVID-19 prevention and control efforts, solidify responsibilities at various levels, and bolster medical security for vulnerable groups such as the older adult, children, the sick, the disabled, and pregnant women in rural areas (19, 20). This approach aims to maximize the health of rural residents and maintain normalcy in production and daily life. Grassroots medical institutions and their staff face escalating responsibilities and tasks (21, 22). The absence of infectious disease specialists at these institutions suggests an area for improvement in the infectious disease control capabilities of medical staff. It is recommended that grassroots medical staff continuously enhance their infectious disease prevention skills to accommodate the growing demands for treatment. Government-led implementation of epidemic prevention strategies and measures should be complemented by regular infectious disease-related training in grassroots hospitals (23). This training should be tailored to meet specific needs and strengthen the medical staff’s preparedness for sudden infectious disease outbreaks and emergency response capacities. Such initiatives will ensure a more effective and proactive approach to the evolving epidemiological landscape (24–26).

## 5 Conclusion

We started from Guangxi, which has a relatively backward economy in China, and explored the changes in the ability of grassroots physicians to prevent and control infectious diseases before and after COVID-19, and analyzed the influencing factors. In the face of the sudden outbreak of the epidemic, it is worth exploring whether areas with poor medical resources have sufficient capacity to fight against the epidemic and ensure the safety of the people. From the research results, it can be seen that the Chinese government at all levels has improved the epidemic prevention and control capabilities of grassroots physicians in underdeveloped areas through a series of measures. In the future, it is still necessary to focus on the development of continuing education and training for doctors, increase the proportion of senior professional titles, and effectively improve the overall epidemic prevention and control level.

## 6 Limitations

Anyway, our research still has certain limitations. Firstly, although our sample size meets the minimum requirement for random sampling research, there are still certain differences in the economic development level of different regions in Guangxi. The score differences caused by these factors also need to be comprehensively considered and analyzed. Secondly, we only selected Guangxi, which is at a relatively low level in the Chinese economy, for the questionnaire survey analysis. The results cannot fully represent the overall situation of underdeveloped areas in the Chinese economy. In the future, we can consider expanding the research object and conducting joint surveys in multiple regions for comprehensive analysis. Finally, since our study period encompassed both pre-and post-COVID-19 eras, the research design was not specifically developed to assess pandemic response capabilities. Consequently, our questionnaire items may not directly address COVID-19-related competencies and might not fully capture the evolution of primary care physicians’ pandemic preparedness. Moving forward, we plan to revise the questionnaire design to incorporate more comprehensive assessment criteria for infectious disease prevention and control, including pandemic-specific capabilities.

## Data Availability

The original contributions presented in the study are included in the article/Supplementary material, further inquiries can be directed to the corresponding author.
