# Supplementary material for: Assessing the evolution of infectious disease preparedness among a province with poor economy in China in the wake of COVID-19
Source: Front Public Health. 2025 May 26;13:1472331. doi: 10.3389/fpubh.2025.1472331 (PMC12146328; doi:10.3389/fpubh.2025.1472331)
Supplement: Supplementary file 1 [file Data_Sheet_1.docx]

Questionnaire Number:

**Survey questionnaire on awareness of infectious disease prevention and control**

**Instructions for filling out the form:**

We conducted this survey to understand the knowledge of grassroots medical staff on infectious disease prevention and control.Our survey has been approved by the Ethics Committee of Guangxi Health Science College (approval number: Z-A20221009). This questionnaire survey is anonymous and the results are only for statistical analysis. We will not disclose any personal privacy information about you. Please rest assured. Please read the following questions carefully and check the corresponding answers. Thank you very much for your support!

**Part I: General Information**

1. Gender: ① Male ② Female

2. Age:_________

3. Highest educational level: ① University ② High school ③ Junior high school ④ Primary school ⑤ None

4. Ethnicity: ① Han chinese ② Chinese Zhuang ethnic group ③ Other

5. Nature of your organization: ① Rural medical institutions ② Individual practicing doctor ③ Technician/Technician ④ Preventive Medicine Doctor ⑤ Other

6. Professional title: ① Primary ② Intermediate ③ Senior ④ No professional title

7. Medical practice location:________

**Part II:**

**Understanding of Statutory Infectious Diseases and Epidemic Reporting**

1. According to the Infectious Disease Prevention and Control law of China, which of the following is classified as Class B infectious diseases

A. Pestis

B. Cholera

C. Leprosy

D. AIDS

E. Rubella

2. According to the report and control measures for Class A infectious diseases, which of the following are classified as Class B infectious diseases

A. SARS, pulmonary anthrax, poliomyelitis and pestis

B. SARS, highly pathogenic avian influenza, AIDS and viral hepatitis

C. Infectious atypical pneumonia and pulmonary anthrax

D. Human infection with highly pathogenic avian influenza, pestis, AIDS and viral hepatitis

E. Pestis, cholera, AIDS and pulmonary anthrax

3. According to the Infectious Disease Prevention and Control law of China, when pulmonary anthrax is discovered, the reporting time limit shall not exceed

A. 24 hours

B. 12 hours

C. 8 hours

D. 6 hours

E. 2 hours

4. Infectious diseases classified as Class B and Class C should be reported online within ( ) hours after diagnosis

A. 2 hours B.6 hours C. 12 hours D. 24 hours

5. According to the Infectious Disease Prevention and Control law of China, which of the following infectious diseases does not require reporting?

A. Hepatitis B Virus carrier B. Mumps C. Syphilis D. Measles

6. Which of the following requirements are correct when filling out an infectious disease report card

A. The Infectious Disease Report Card should be printed uniformly on A4 paper and filled out with a pen or ballpoint pen

B. When patients suffer from two or more infectious diseases at the same time, we can fill out the same card

C. When the patient's disease diagnosis changes, make corresponding modifications on the original report card

D. When the infectious disease registration book has been filled out, the infectious disease report card can be omitted

7. At present, the reporting time limit for human H7N9 avian influenza cases in China is (), and the reporting time limit for human avian influenza cases is ().

A. 24 hours, 6 hours

B. 2 hours, 6 hours

C. 2 hours, 24 hours

D. 24 hours, 24 hours

8. Medical institutions should implement an infectious disease () system, and for infectious disease patients or suspected infectious disease patients, they should be guided to relatively isolated triage points for initial diagnosis.

1. Pre-examination and triage

B. Isolation and disinfection

C. Classification and isolation

D. Designated location and isolation

9. According to the Infectious Disease Prevention and Control law of China, the carrier of the disease source refers to ().

A. People who come into contact with patients with infectious diseases

B. People who come into contact with pathogens

C. A person who is infected with a pathogen without clinical symptoms but can eliminate the pathogen

D. People who are infected with pathogens and have clinical symptoms can also eliminate pathogens

**Relevant Cognition of Infectious Disease Knowledge**

1.Which of the following disinfectants can quickly kill Mycobacterium tuberculosis?

A. 5% Lime Acid Solution

1. 01% peroxyacetic acid
2. 75% alcohol
3. Which of the following is not a route of transmission for hepatitis C?

A. The route of blood transfusion or blood products

B. Fecal oral route

C. Injection route

D. Mother to child transmission

E. Close contact

3. Which of the following treatment principles for acute and chronic hepatitis is incorrect?

A. We should strengthen exercise to enhance physical fitness

B. Take appropriate liver protective drugs

C. Avoid alcohol

D. Do not take liver damaging drugs

E. Get enough rest

4. Which antibody can protect human body from hepatitis B virus infection?

A. Anti-HBs

B. Anti-HBe

C. Anti-HBc IgM

D. Anti-HBc IgG

E. DNA antibody

5.Which is the key step for treating cholera?

A. Antibacterial drugs

B. Inhibition of intestinal mucosal secretion drugs

C. Complications treatment

D. Supplement liquids and electrolytes

6. Which of the following is incorrect in describing the stool characteristics of cholera patients?

A. Wash meat water sample

B. Rice swill water sample

C. Mud sample

D. Water sample

E. Purulent and bloody stool

7. Currently, the main pathogens causing hand-foot-and-mouth disease in China are ()

A. EV72 and Cox16

B. EV71 and Cox16

C. Eko virus

D.CoxA4

E.CoxB2

8. Which of the following statements about epidemic cerebrospinal meningitis is incorrect?

A. When sending specimens of suspected patients with meningitis for testing, attention should be paid to insulation and rapid submission

B. Epidemic cerebrospinal meningitis is highly contagious and belongs to Class A infectious diseases in the list of legally recognized infectious diseases

C. The common type accounts for 90% of all cases

D. Carriers are an important source of infection for the spread of meningitis

E. Susceptible individuals who have come into contact with patients with meningitis can take prophylactic sulfonamide drugs

9. What are the transmission routes of Japanese encephalitis?

A. Respiratory transmission through droplets

B. Fecal contamination of water sources and oral transmission of food

C. Flies as a vector contaminate food and transmit it orally

D. Mosquito bites carrying viruses can spread through the skin and blood

E. Spread through blood and bodily fluids

10. Which of the following is not the mode of transmission of HIV virus?

A. Sexual contact transmission

B. Blood products

C. Mother to child transmission

D. Damaged skin contaminated

E. Respiratory transmission

11. What is the preferred treatment for Pneumocystis carinii pneumonia complicated by AIDS?

A. Penicillin

B. Fluconazole

C. Ganciclovir

D. Spiramycin

E. Compound sulfamethoxazole (compound sulfamethoxazole)

12. Which of the following is incorrect regarding the epidemiology of rabies?

A. The main source of rabies transmission in developed countries is wild animals

B. The main source of rabies transmission in China is infected dogs

C. Animals with normal appearance do not cause rabies

D. The virus is mainly transmitted through bites

E. It can also be transmitted through the respiratory tract

**Part III: Knowledge and Skill Requirements for Infectious Disease Prevention and Control Work**

1. Have you ever treated or discovered patients with infectious diseases?

①Yes ②No

2. Which type of infectious disease do you mainly treat in your daily consultations?

① Respiratory infectious diseases

② Infectious disease of digestive tract

③ Blood and body fluid infectious diseases

④ Insect borne diseases

⑤ Other

3. For patients suspected of having infectious diseases, what are your main responsibilities (multiple choice)

① Guide referral ② Disinfection and isolation ③ Assist in epidemic investigation and tracking ④ Report ⑤ Other

4. For patients with chronic infectious diseases, your main responsibilities include:

① Regularly prescribe medication

② Health Education

③ Family isolation guidance

④ Other

1. What are your main difficulties in infectious disease prevention and control work? (Multiple Choice)

① Pre-diagnosis of infectious diseases

② Diagnosis and differential diagnosis of infectious diseases

③ Inspection Method

④ Therapeutic medication

⑤ Indications for referral

⑥ Epidemic reporting

⑦ Disinfection and isolation

⑧ Vaccination

⑨ Health guidance

1. Have you received training on infectious disease prevention and control, as well as epidemic reporting?

① Correct ② Deny ③ Can't remember clearly

As for ①Yes, then the last time I received training was in years

7. If you want to receive training, what kind of training do you need the most? (Multiple Choice)

① Pre-diagnosis of infectious diseases

② Diagnosis and differential diagnosis of infectious diseases

③ Inspection Method

④ Therapeutic medication

⑤ Indications for referral

⑥ Epidemic reporting

⑦ Disinfection and isolation

⑧ Vaccination

⑨ Health guidance

8. Do you wish to receive training related to infectious diseases?

①Yes

②No
