# Supplementary material for: Assessing the evolution of infectious disease preparedness among a province with poor economy in China in the wake of COVID-19
Source: Front Public Health. 2025 May 26;13:1472331. doi: 10.3389/fpubh.2025.1472331 (PMC12146328; doi:10.3389/fpubh.2025.1472331)
Supplement: Supplementary file 2 [file Data_Sheet_2.docx]

Questionnaire Number:

**Survey questionnaire on awareness of infectious disease prevention and control**

**Instructions for filling out the form:**

We conducted this survey to understand the knowledge of grassroots medical staff on infectious disease prevention and control.Our survey has been approved by the Ethics Committee of Guangxi Health Science College (approval number: Z-A20221009). This questionnaire survey is anonymous and the results are only for statistical analysis. We will not disclose any personal privacy information about you. Please rest assured. Please read the following questions carefully and check the corresponding answers. Thank you very much for your support!

**Part I: General Information**

1. Gender: ① Male ② Female

2. Age:___________

3. Highest educational level: ① University ② High school ③ Junior high school ④ Primary school ⑤ None

4. Ethnicity: ① Han chinese ② Chinese Zhuang ethnic group ③ Other

5. Nature of your organization: ① Rural medical institutions ② Individual practicing doctor ③ Technician/Technician ④ Preventive Medicine Doctor ⑤other

6. Professional title: ① Primary ② Intermediate ③ Senior ④ No professional title

7. Medical practice location:_________

**Part II****:**

**Understanding of Statutory Infectious Diseases and Epidemic Reporting**

1. According to the laws of the People's Republic of China on the Prevention and Control of Infectious Diseases, the infectious diseases managed are divided into several categories and types, such as ( )

A. 2 in Class A, 26 in Class B, and 11 in Class C

B. 2 in Class A, 25 in Class B, and 10 in Class C

C. 2 in Class A, 27 in Class B, and 11 in Class C

2. Which of the following belongs to Class A infectious diseases

A. Cholera and pestis

B. Measles and novel coronavirus pneumonia

C. Pulmonary anthrax and SARS

3. When discovering Class A and Class A managed infectious diseases, suspected patients, or other infectious diseases and unknown disease outbreaks, the infectious disease report card should be reported online within ( )

A. 2 hours

B. 6 hours

C. 12 hours

D. 24 hours

4. Infectious diseases classified as Class B and Class C should be reported online within ( ) hours after diagnosis

A. 2 hours

B. 6 hours

C. 12 hours

D. 24 hours

5. In china, if the network reporting personnel of the responsible reporting unit discover 3 or more cases of the same acute infectious disease in a natural village, street, or collective unit (place) within one day, or 5 or more cases within one week, they should report the information directly to the network within ( ) hours, and at the same time report to the local county (district) level disease prevention and control institution by phone

A. 1 hour

B. 2 hours

C. 6 hours

D. 24 hours

6. When a patient has two infectious diseases at the same time, how many infectious disease report cards should you fill out?

A. 1

B. 2

7. Which of the following diseases does not need to be filled in as "acute" or "chronic" when filling out the infectious disease report card?

A. Hepatitis

B. Typhoid and paratyphoid fever

C. Schistosomiasis

8.What type of infectious disease does human infection with highly pathogenic avian influenza belong to? According to Chinese law, how should it be managed during an outbreak?

A. Belonging to Class B infectious disease, it is managed according to Class A infectious disease during epidemic

B. Belonging to Class B infectious disease, it is managed according to Class B infectious disease during epidemic

C. Belonging to Class C infectious disease, it is managed according to Class B infectious disease during epidemic

9.According to the Chinese Infectious Disease Prevention and Control law, which of the following does not belong to the transmission route of infectious diseases??

A. Airborne transmission

B. Droplet transmission

C. Contact transmission

D. Water transmission

**Relevant Cognition of Infectious Disease Knowledge**

1. How is tuberculosis transmitted?

A. Digestive tract

B. Respiratory tract (airborne droplet transmission)

C. Blood transfusion

2. Which of the following epidemiological data is the least important for patients with hepatitis B virus?

A. Blood transfusion history

B. Injection history

C. Dietary hygiene

D. Vaccination history

3. The most important treatment measure for early acute viral hepatitis is ()

A. Bed rest

B. Hepatoprotective drugs

C. Antiviral drugs

D. Vitamin drugs

4. HBsAb (+), HBeAb (+), HBcAb (+), that means ( )

A. Highly contagious, acute attacks in early or chronic infected individuals

B. Further testing of virus replication is needed

C. In the recovery period of acute infection, where immunity has been developed

5. How is cholera transmitted?

A. Digestive tract

B. Respiratory tract (airborne droplet transmission)

C. Blood transfusion

6. The daily frequency of bowel movements for patients with diarrhea is

A. ≥ 1

B. ≥ 2

C. ≥ 3

D. ≥ 4

7. EV71 and other viruses are the main pathogens causing hand-foot-and mouth disease, mainly infecting

A. Children under 10 years old

B. Teenagers between 10-15 years old

C. Young adults between 20-40 years old

D. Elderly people over 60 years old

8. The isolation method that should be adopted for epidemic cerebrospinal meningitis (meningitis) is:

A. Strict isolation

B. Digestive tract isolation

C. Respiratory tract isolation

D. Blood isolation

9.What are the transmission routes of Japanese encephalitis?

A. Digestive tract transmission

B. Respiratory tract (airborne droplet transmission)

C. Contact transmission

D. Mosquito bite transmission

10.Which of the following behavior is most likely to cause the spread of HIV?

A. Sharing toilets with people living with HIV

B. General contact with people living with HIV

C. Mothers with HIV breastfeeding their babies

11. The time of taking preventive medicine after AIDS exposure should not exceed ( )

A. 24 hours

B. 36 hours

C. 48 hours

D. 72 hours

12. Do you think rabies is currently?

A. Treatable

B. Treatable and preventable

C. Preventable but incurable

**Part III: Knowledge and Skill Requirements for Infectious Disease Prevention and Control Work**

1. Have you ever treated or discovered patients with infectious diseases?

①Yes ②No

2. Which type of infectious disease do you mainly treat in your daily consultations?

① Respiratory infectious diseases

② Infectious disease of digestive tract

③ Blood and body fluid infectious diseases

④ Insect borne diseases

⑤ Other

3. For patients suspected of having infectious diseases, what are your main responsibilities (multiple choice)

① Guide referral ② Disinfection and isolation ③ Assist in epidemic investigation and tracking ④ Report ⑤ Other

4. For patients with chronic infectious diseases, your main responsibilities include:

① Regularly prescribe medication

② Health Education

③ Family isolation guidance

④ Other

1. What are your main difficulties in infectious disease prevention and control work? (Multiple Choice)

① Pre-diagnosis of infectious diseases

② Diagnosis and differential diagnosis of infectious diseases

③ Inspection Method

④ Therapeutic medication

⑤ Indications for referral

⑥ Epidemic reporting

⑦ Disinfection and isolation

⑧ Vaccination

⑨ Health guidance

1. Have you received training on infectious disease prevention and control, as well as epidemic reporting?

① Correct ② Deny ③ Can't remember clearly

As for ①Yes, then the last time I received training was in years

7. If you want to receive training, what kind of training do you need the most? (Multiple Choice)

① Pre-diagnosis of infectious diseases

② Diagnosis and differential diagnosis of infectious diseases

③ Inspection Method

④ Therapeutic medication

⑤ Indications for referral

⑥ Epidemic reporting

⑦ Disinfection and isolation

⑧ Vaccination

⑨ Health guidance

8. Do you wish to receive training related to infectious diseases?

①Yes

②No
